# Kidney Renin Release under Hypoxia and Its Potential Link with Nitric Oxide: A Narrative Review

**DOI:** 10.3390/biomedicines11112984

**Published:** 2023-11-06

**Authors:** Weiwei Kong, Yixin Liao, Liang Zhao, Nathan Hall, Hua Zhou, Ruisheng Liu, Pontus B. Persson, Enyin Lai

**Affiliations:** 1 Kidney Disease Center of First Affiliated Hospital, Zhejiang University School of Medicine, Hangzhou 310003, China; weiweikong@zju.edu.cn; 2 Department of Physiology, School of Basic Medical Sciences, Zhejiang University School of Medicine, Hangzhou 310003, China; 3 Department of Obstetrics and Gynaecology, Nanfang Hospital, Southern Medical University, Guangzhou 510515, China; yinxin1225@126.com; 4 Department of Nephrology, Children’s Hospital, Zhejiang University School of Medicine, National Clinical Research Center for Child Health, Hangzhou 310052, China; liang.zhao@zju.edu.cn; 5 Department of Molecular Pharmacology & Physiology, Morsani College of Medicine, University of South Florida, Tampa, FL 33612, USA; nathanhall@usf.edu (N.H.); ruisheng@usf.edu (R.L.); 6 Department of Nephrology, Shengjing Hospital of China Medical University, Shenyang 110004, China; huazhou_cmu@163.com; 7 Institute of Translational Physiology, Charité–Universitätsmedizin Berlin, 10117 Berlin, Germany; pontus.persson@charite.de

**Keywords:** renin, hypoxia, HIFs, juxtaglomerular cells, nitric oxide

## Abstract

The renin–angiotensin system (RAS) and hypoxia have a complex interaction: RAS is activated under hypoxia and activated RAS aggravates hypoxia in reverse. Renin is an aspartyl protease that catalyzes the first step of RAS and tightly regulates RAS activation. Here, we outline kidney renin expression and release under hypoxia and discuss the putative mechanisms involved. It is important that renin generally increases in response to acute hypoxemic hypoxia and intermittent hypoxemic hypoxia, but not under chronic hypoxemic hypoxia. The increase in renin activity can also be observed in anemic hypoxia and carbon monoxide-induced histotoxic hypoxia. The increased renin is contributed to by juxtaglomerular cells and the recruitment of renin lineage cells. Potential mechanisms regulating hypoxic renin expression involve hypoxia-inducible factor signaling, natriuretic peptides, nitric oxide, and Notch signaling-induced renin transcription.

## 1. Introduction

The renin–angiotensin system (RAS) is a hormonal system responsible for blood pressure homeostasis and electrolyte balance. This systemic RAS consists of several key components: renin, angiotensinogen (AGT), angiotensin I (Ang I), angiotensin-converting enzymes (ACEs), angiotensin II (Ang II), angiotensin II type 1 receptors (AT1Rs), and angiotensin II type 2 receptors (AT2Rs) [1,2]. Renin metabolizes AGT, liberating Ang I. ACEs, which are released from endothelial cells, convert Ang I to Ang II. Then, Ang II acts on two types of receptors, including AT1Rs and AT2Rs. The actions of Ang II on AT1Rs lead to increased sodium retention, vasoconstriction, stimulation of thirst and desire for salt, increased sympathetic nervous system activity, and aldosterone release. Ang II actions on AT2Rs are counter to those on AT1Rs, where AT2R stimulation leads to anti-inflammatory, antifibrotic, and vasodilatory effects. In addition to the classic axis, another axis through ACE2/Ang-(1–7)/MAS has been found. ACE homolog ACE2 can form Ang-(1–7) directly or indirectly from either the decapeptide Ang I or from Ang II. By acting through the receptor MAS, Ang-(1–7) promotes vasodilation, antiproliferation, and antihypertrophy [3,4,5]. Apart from the systemic RAS, local RASs have been found in various organ systems. Local RASs exhibit multiple physiological effects in addition to and distinct from those of the circulating RAS. In addition to hemodynamic actions, multiple and novel functions of local RASs have been found, including the regulation of cell growth, differentiation, proliferation and apoptosis, reactive oxygen species (ROS) generation, tissue inflammation and fibrosis, and hormonal secretion [4,5].

Hypoxia is a state that arises when the cellular demand for molecular oxygen exceeds supply. It often occurs in tissue and organs with microcirculation injury and hypoperfusion [6]. Under some hypoxia conditions, the RAS is activated and the level of Ang II increases [7,8,9]. In return, Ang II binding on AT1Rs increases the constriction of both afferent and efferent arterioles, which can aggravate hypoxia [10]. Oxidative stress is a condition that results in the excessive production of oxygen radicals beyond the antioxidant capacity. Both Ang II and hypoxia can induce oxidative stress, which decreases oxygen supply and increases oxygen demand. Finally, the progression of renal injury by oxidative stress further aggravates hypoxia and the activation of RAS. The complexity of the relationship between RAS, hypoxia, and oxidative stress is a vicious cycle [11]. The amount of renin in the bloodstream is a key step in determining Ang II levels and RAS activity [12]. Thus, the pathophysiological change in renin secretion under hypoxia plays a crucial role in the complex correlation between the RAS and hypoxia. To provide the latest evidence for future research and practice, this study critically reviewed the role of kidney renin under hypoxia. Two electronic databases, PubMed and Web of Science, were searched and the main keywords were ‘hypoxia’ AND ‘renin’.

## 2. Renin Expression in Kidneys

From the embryonic stage, renin expression is located in renin precursor cells (RPCs) [13,14]. During kidney development, RPCs gradually disappear, mostly differentiating into renal vascular smooth muscle cells (VSMCs), mesangial cells, and interstitial pericytes. At the same time, the expression of renin in tubular cells also disappears. After kidney development is complete, only a small number of renin-expressing cells are maintained and restricted to the juxtaglomerular apparatus (JG) in adult mammals. They synthesize and release renin in response to a decrease in renal perfusion pressure, a decrease in the concentration of sodium chloride in the macula densa, or activation of β adrenergic receptors [15,16]. The number of JG cells is quite small in adult mammals, accounting for only about 0.01% of total kidney cells. Under normal circumstances, the release of renin by those few cells generally suffices for maintaining blood pressure and fluid–electrolyte balance [17,18]. Although renin-expressing cells are classically regarded as JG cells in adults, some studies have established that an increase in renin-expressing cells can be derived from the renin lineage cells. This process is termed recruitment. The renin lineage cells in kidneys are not fully differentiated but retain a degree of developmental plasticity or molecular memory, allowing them to re-express renin. Under homeostatic threats, the renin lineage cells like VSMCs, mesangial cells, and interstitial peritubular pericytes are transformed to synthesize renin [19,20,21].

## 3. The Change in Renin under Hypoxia

Oxygen is a significant microenvironmental factor that acts as a terminal electron acceptor in oxidative phosphorylation reactions to produce adenosine triphosphate (ATP). Hypoxia is an insufficient supply of oxygen to tissues, which results in abnormal cell metabolism and function [22,23]. According to the etiology of hypoxia, hypoxia can be classified as hypoxemic hypoxia, anemic hypoxia, stagnant hypoxia, or histotoxic hypoxia. According to the time of hypoxia, hypoxia can also be divided into chronic hypoxia and acute hypoxia. Chronic hypoxia includes sustained hypoxia and intermittent hypoxia [24]. Here, we summarise the changes in systemic renin under different hypoxia conditions (Table 1). 

For hypoxemic hypoxia, studies have confirmed that plasma renin activity (PRA) increases after acute hypoxia treatment under different oxygen concentrations. PRA increases from 2.3 ± 0.4 ng/mL/h to 4.9 ± 0.8 ng/mL/h after 8% oxygen breathing for 20 min and increases from 2.8 ± 0.4 ng/mL/h to 8.4 ± 1.8 ng/mL/h after 5% oxygen breathing for 20 min [25]. A 12% oxygen breathing treatment for 20 min in rats increases PRA from 3.08 ± 0.68 ng/mL/h to 8.36 ± 1.8 ng/mL/h [26]. Sustained hypoxia (10% oxygen) leads to a decrease in renal renin gene expression to 76% of that of a control after two weeks of treatment and 49% of a control after four weeks of treatment [27,28]. Intermittent hypoxia is composed of hypoxia–normoxia cycles. Hypoxia–normoxia cycles are controlled by individually ventilated cages, which can rapidly change the fraction of inspired oxygen (FiO_2_) in seconds [29]. In Fletcher’s research, the hypoxia–normoxia cycle was 2 min per cycle and the lowest FiO_2_ level reached 2% or 3%. After 35 days of intermittent hypoxia treatment, PRA increased about fourfold compared with the control group [30]. Saxena’s team set a longer intermittent hypoxia cycle of 6 min and the lowest FiO_2_ level was 10%. The PRA also increased after 1 day and 7 days of intermittent hypoxia treatment [31]. In summary, acute hypoxemic hypoxia or intermittent hypoxemic hypoxia both induce an increase in renin activity but sustained hypoxemic hypoxia negatively regulates renin activity. 

Obstructive sleep apnea (OSA) is a common type of intermittent hypoxemic hypoxia in clinics that is characterized by recurrent episodes of oxygen desaturation and reoxygenation [32]. A meta-analysis conducted on 13 studies found that elevated aldosterone levels were observed in OSA patients with hypertension compared to normotensive OSA patients. Hypertensive disorders are strongly linked with an overactive RAS because the activation of RAS can regulate the body’s hemodynamic equilibrium, circulating volume, and electrolyte balance. The activation of RAS has been implicated in playing a pathophysiological role in the relationship between OSA and hypertension, particularly resistant hypertension [33]. 

Another special type of hypoxemic hypoxia occurs at high altitudes. With increasing altitude, the partial pressure of oxygen in the ambient air decreases. Humans ascending to high altitudes inhale fewer oxygen molecules per breath [34]. The effect on plasma renin activity depends on the time spent at high altitudes. Two studies have moved the objects of observation from sea level to high altitudes and have tested the change in renin at different time points. A short time at high altitudes causes a decrease in RAS, which seems to be protective against plasma Ang II and could lead to vasoconstriction and sodium retention. After a prolonged stay at high altitudes, plasma renin activity increases but remains reduced compared to at sea level. The different change in renin at high altitudes compared to classical hypoxemic hypoxia may be related to complex modifications in systemic vascular dysfunction. In addition to the exposure to hypoxia, high altitudes cause a significant increase in aortic stiffness, blood pressure, heart rate, and cardiac output. RAS is not to be the determining factor for vascular changes because the inhibition of RAS did not show any difference in parameters reflecting the viscoelastic properties of large arteries at high altitudes [35,36]. Both hypoxia and systemic vascular dysfunction can influence a change in RAS, which may be the reason for a different change in renin at high altitudes compared to classical hypoxia. Another study compared high-altitude natives and sea-level natives. It found that the level of PRA was higher in high-altitude natives compared with sea-level natives [37]. In this study design, the higher PRA may be influenced by hypoxia but is also affected by cardiopulmonary maladaptation, race, diet, and lifestyle.

Erythrocytes are essential for the delivery of oxygen to organs. Anemia causes a whole-body oxygen shortage, which belongs to anemic hypoxia [38]. Kenichiro Miyauchi’s team utilized inherited super anemic mutant (ISAM) mice to modulate anemia-related hypoxia. Under anemic hypoxia, the levels of renin expression increased in the kidneys [39]. Exposure to carbon monoxide is one kind of histotoxic hypoxia. Kramer et al. found that exposure to carbon monoxide increased PRA approximately three to four times and boosted renin mRNA levels approximately two times compared to the control group [40]. Stagnant hypoxia is usually accompanied by changes in hemodynamics, which may play a more dominant role in regulation than hypoxia. Thus, it is hard to evaluate the renin changes that are contributed to by the insufficient supply of oxygen under the condition of stagnant hypoxia.

**Table 1:** The change in renin under hypoxia.

			Change in Renin Activity/Expression	Reference
**Acute hypoxemic hypoxia**				
20 min	Beagle dogs	5 and 8% O_2_	Increased	[25]
20 min	SD rats	12% O_2_	Increased	[26]
**Chronic sustained hypoxemic hypoxia**				
2/4 w	Wistar rats	10% O_2_	Decreased	[27,28]
**Chronic intermit hypoxemic hypoxia**				
35 d	Wistar rats	2–3% O_2_ 2 min/cycle	Increased	[30]
1 d	SD rats	10% O_2_ 6 min/cycle	Increased	[31] [31]
7 d	SD rats	10% O_2_ 6 min/cycle	Increased	
**Special type of hypoxemic hypoxia**				
Obstructive sleep apnea			Increased	[33]
High altitude natives (vs sea level natives)			Higher	[37]
6 days stay at high altitude (vs basal at sea level)			Decreased	[35]
Acute exposure to high altitudes (vs basal at sea level)			Decreased	[36]
2 weeks stay at high altitudes (vs basal at sea level)			Decreased	[36]
**Anemic hypoxia**				
Inherited super anemic mutant mice			Increased	[39]
**Histotoxic hypoxia**				
6 h	SD rats	0.1% Carbon monoxide	Increased	[40]

## 4. Sources of Renin under Hypoxia 

**Figure 1:**
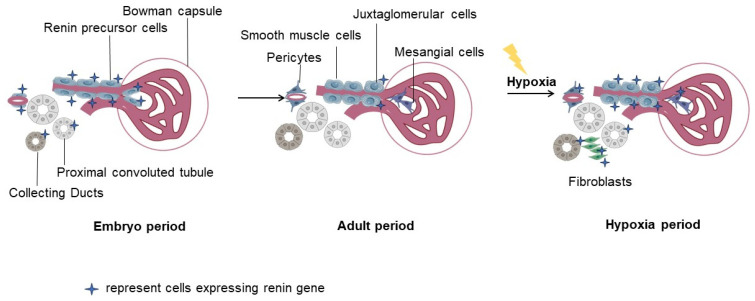
The expression of renin in kidneys under hypoxia. From the embryonic stage, renin expression is located along vascular and around collecting ducts and proximal tubules. As the kidneys develop, renin precursor cells gradually disappear and most of them differentiate into intrinsic renal cells (like pericytes, mesangial cells, and smooth muscle cells). Renin-expressing tubular cells in the embryonic stage also disappear in the adult period. After kidney development is complete, only a small number of renin-expressing cells are maintained and are restricted to the juxtaglomerular (JG) apparatus in adult mammals. The expression of renin increases under hypoxia, which is observed in both JG cells and other intrinsic renal cells. The descendants of renin precursor cells can transform into their pre-differentiated states to re-express the renin gene. Also, renin-expressing tubular cells in the embryonic stage can re-express renin in response to hypoxia.

This part summarizes two sources of renin expression under hypoxia, including the activation of JG cells and the recruitment of renin lineage cells (Figure 1). 

The activation of JG cells under hypoxia: JG cells store renin in dense core secretory granules and can become hypergranulated when renin secretion increases [41]. To study the change in hypoxia on JG cells, Goldfarb first used a hypoxia chamber but found no changes in the morphology of JG cell granularity under the hypoxia condition of one-half atmospheric pressure for 12 h [42]. Furthermore, Oliver et al. found that hypoxia-treated rats exhibited poor intake of food and sodium compared to control rats. Since serum sodium levels can influence renin secretion, they improved the research design by adding sodium supplements. In Olivers study, a supplemental injection of sodium chloride was used daily to avoid the effect of sodium deprivation. The hypoxia condition was constructed by maintaining an oxygen content of 7% to 8%. Finally, they verified that hypoxia induces the hypergranulation of JG cells [43]. The in vivo preliminarily experiment revealed that hypoxia leads to the granularity of JG cells, but the in vitro experiment did not present similar results. In the primary culture of renal JG cells, hypoxia treatment (1% or 3% oxygen for 6 or 20 h) did not affect renin activity [40]. The difference between in vivo and in vitro studies indicates that the link between renin secretion and hypoxia may be indirect, reflecting the demand for some kind of systemic signaling link. The sympathetic nervous system may be one of the potential linking mechanisms. Renin secretion is the downstream effector of the sympathetic nervous system, and afferent nerve signals are required during the stimulation process. An in vitro culture of JG cells is a state of renal denervation, which is not regulated by the sympathetic nervous system [44]. Circulating catecholamine may be another potential medium related to hypoxia and renin secretion. Previous evidence showed that circulating catecholamines such as noradrenaline and adrenalin are stimulated under some hypoxia conditions and catecholamine-induced receptor activation significantly stimulates both renin secretion and gene expression. Local renal factors, such as the renal baroreceptor mechanism or the macula densa mechanism, may be a step in the systemic signaling link, but are not yet clearly understood [45,46,47,48,49]. 

Re-expression of renin under hypoxia: RPCs and their descendants are the center of nephrogenesis. With kidney development, RPCs gradually disappear and most of these RPCs differentiate into renal VSMCs, mesangial cells, interstitial pericytes, and renal tubular cells. Such renin lineage cells in adults retain their ability to transdifferentiate into the original state to re-express renin [50]. Renin lineage cells transformed into their pre-differentiated state to re-express the renin gene is a process known as recruitment [51].

The recruitment process in renin lineage cells was found in anemic hypoxia. Miyauchi et al. found that renin activity increases in an anemia model of ISAM mice. As expected, the expression of renin 1 structural (*Ren1*) mRNA was consistently detected in JG cells in both the ISAM mice and the control mice. But, the *Ren1* expression in renal interstitial cells could be detected in ISAM mice, but not in control mice. In situ hybridization analysis found that interstitial cells with *Ren1* positive were fibroblasts with the characteristic of being positive pericyte markers [39]. It was preliminarily shown that renin-expressing fibroblasts were transdifferentiated from recruitment pericytes. Unilateral ureteral obstruction (UUO) is a classic model of chronic obstructive kidney disease, with the characteristic of tubulointerstitial fibrosis. Tubulointerstitial fibrosis affects oxygen diffusion and supply, leading to tissue hypoxia [52]. The recruitment process was also found in the UUO model. RenCreER (Ren1cCreERxRs-tdTomato) transgenic mice were applied to the fate map of *Ren1^+^* cells in the UUO model, in which *Ren1^+^* progenitors were permanently labeled during a period of tamoxifen induction. There was only a marginal increase in *Ren1^+^* cells in the JG areas. However, the number of *Ren1^+^* cells in interstitial areas increased significantly from day 7 after UUO. The labeled *Ren1^+^* cells initially co-expressed the pericyte markers and appeared around peritubular capillaries. On day 14 post-UUO, the majority of labeled cells were away from blood vessels and transdifferentiated into myofibroblasts. *Ren1^+^* cells around interstitial areas in a UUO model possibly participated in vessel remodeling in the early period and ultimately underwent transitions into myofibroblast-like cells, which might favor fibrosis rather than repair. Interestingly, the recruitment of *Ren1^+^* cells in UUO cannot retain renin protein, which was indicated by the fact that renin protein staining was negative in interstitial areas [53]. Similarly, another study found that recruitment pericytes in interstitial areas cannot store renin [54]. Renin storage is a crucial part of renin secretion. Thus, it is currently recognized that recruitment cells can express the renin gene, but is unknown whether the recruitment cells can lead to renin secretion. Future studies are needed to give us the answer. 

## 5. Potential Mechanisms Regulating Renin under Hypoxia 

**Figure 2:**
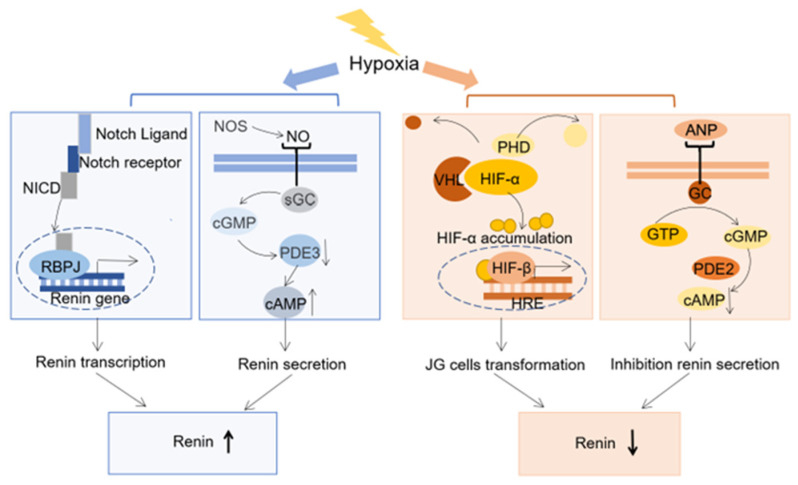
Possible mechanism regulating renin expression under hypoxia. Notch signaling is activated under hypoxia and then the Notch intracellular domain (NICD) translocates into nuclear and binds with the co-effector recombination signal sequence binding protein J kappa (REBPJ) to form the transcription complex. The transcription complex has a specific binding sequence and binds with the promoter of the renin gene, which finally induces renin transcription. Nitric oxide (NO) is essential to regulate renin secretion. Hypoxia can promote NO production by increasing nitric oxide synthase (NOS), especially neuronal-type NOS (nNOS), transcription. Increased NO can stimulate soluble guanylate cyclase (sGC) and increase cyclic guanosine monophosphate (cGMP). Then, cGMP-mediated inhibition of phosphodiesterase (PDE) 3 prevents cAMP degradation and stimulates renin secretion. The activation of hypoxia-inducible factor (HIF) signaling is a hypoxia protection mechanism. Under normoxic conditions, HIF-α subunits are hydroxylated by the prolyl 4-hydroxylase domain (PHD) and then degraded by the von Hippel–Lindau tumor suppressor protein (VHL). Under hypoxia, the hydroxylation of HIF-α byVHL is inhibited and HIF-α/HIF-β heterodimers bind to the HIF-responsive element (HRE), thereby promoting the transcription of downstream genes. The activation of HIF signaling in JG cells leads to a reprogramming of cells against hypoxia by increasing erythropoietin (EPO) secretion and reducing renin secretion. Atrial natriuretic peptide (ANP) is hypoxia-responsive and has an inhibitory role for renin secretion.its mechanism starts from binding with the guanylyl cyclase (Gc) receptor, then stimulates the cGMP/PDE2 pathway, and subsequently degrades the cyclic adenosine monophosphate (cAMP).

Here, we summarized the potential mechanisms for regulating renin expression or secretion under hypoxia. Notch signaling and nitric oxide (NO) are potential up-regulation mechanisms, while hypoxia-inducible factors (HIFs) and natriuretic peptides are efforts to downregulate renin (Figure 2).

Hypoxia-inducible factors (HIFs) regulating renin expression: HIFs are key transcription factors in the response to hypoxia [55]. HIFs consist of one HIF-α subunit and one HIF-β subunit. There are three isoforms of the HIF-α subunit (HIF-1α, HIF-2α, and HIF-3α), among which HIF-1α and HIF-2α are critical in the response to hypoxia [56,57]. Under normoxic conditions, HIF-α subunits are hydroxylated by the prolyl 4-hydroxylase domain (PHD) and then degraded by the von Hippel–Lindau tumor suppressor protein (VHL) [58,59]. Under hypoxia, the hydroxylation of HIF-α is inhibited and HIF-α combines with HIF-β to form HIF-α/HIF-β heterodimers. Subsequently, the HIF-α/HIF-β heterodimers bind to the HIF-responsive element (HRE), thereby promoting the transcription of downstream genes [60,61]. Recently, researchers found that HIF-α accumulation under normoxia leads to a change in hormone expression in JG cells. The deletion of both PHD2 and PHD3 upregulates HIF-α accumulation, which is accompanied by reduced renin expression and promoted erythropoietin (EPO) expression in both JG cells and interstitial cells [54]. Similarly, HIF overexpression induced by PHD inhibitors also increases EPO levels in peripheral blood [62,63]. Furthermore, Kurt et al. developed a mouse model involving the specific deletion of VHL in renin-expressing cells. The mouse model shows downregulated renin activity in the baseline condition. Also, the mice-specific deletion of VHL also has an attenuated expansion in renin-expressing cells, even under the stimulation of the RAS (a low-salt diet combined with an ACE inhibitor). The deletion of VHL in renin-expressing cells activates EPO expression [64]. 

The switch of hormone expression in JG cells under the HIF signaling activation is associated with changes in morphological and gene expression profiles. Electron microscopy reveals that control JG cells have cuboid-like morphological features with an accumulation of prominent electron-dense renin storage vesicles. However, JG cells with the specific deletion of VHL have flat and elongated morphological features with no classical electron-dense granules. Additionally, gene expression profiles show a loss of typical markers of renin cells and an increase in fibroblast markers in pVHL-deficient JG cells [65,66]. Till now, both pharmaceutical-treated mice and transgenic mice with HIF-α accumulation under normoxia have shown increased expression of EPO and decreased expression of renin under normoxia. Such changes in renin-expressing cells help alleviate hypoxia because increased EPO expression may enhance oxygen delivery and decreased renin expression can lead to vasorelaxation. This change is in accordance with the protective role of HIFs in hypoxia.

Potential role of Notch signaling in renin expression: The Notch signaling pathway is critical for normal cell proliferation and differentiation. It is composed of receptors, ligands, and the final common effector. The binding of the Notch ligand to its cellular receptor causes the latter to cleave and release the Notch intracellular domain (NICD), which is subsequently translocated into the nucleus. In the nucleus, the NICD binds to the transcription factor recombination signal sequence binding protein J kappa (RBPJ), and then RBPJ as a transcription factor induces downstream gene transcription [67,68].

We supposed a potential link between Notch and renin expression under hypoxia by reviewing previous studies. Firstly, the link between Notch signaling and hypoxia has been reported. Notch signaling is activated under hypoxia, which is supported by the increased expression of NICD and the downstream gene of Notch signaling under hypoxia [69]. In addition, the promotion role of Notch signaling in renin expression has also been reported. The deletion of *RBPJ* in renin lineage cells reveals a significant decrease in the number of renin-positive cells [70]. Also, the expression of two crucial genes that indicate the endocrine phenotype of JG cells, Ren1 and aldo-keto reductase family 1, and member B7 (Akr1b7), substantially diminishes after *RBPJ* deletion [71]. Further, renin gene transcription can be promoted by Notch signaling. As revealed by chromatin immunoprecipitation, the final common effector of Notch signaling RBPJ can bind with the promoter of the renin gene [72]. Furthermore, the mutation of four core nucleotides at the RBPJ binding site of the renin promoter is sufficient to suppress renin expression [71]. Through the above review, an indirect link between Notch signaling and renin expression under hypoxia has been revealed. We indicate that Notch signaling is activated under hypoxia; then, NICD/RBPJ forms the transcription complex and binds with the promoter of the renin gene, which finally induces renin transcription. Jagged1 is one of the five cell surface ligands of the Notch signaling pathway. The classic Jagged1-Notch interaction provokes a cascade of proteolytic cleavages, which transport the NICD into the nucleus [73]. However, the specifically conditional deletion of Jagged1 within renin-expressing cells does not result in downregulated renin expression in JG cells [74]. This indicates that renin transcription regulated by Notch signaling is not dependent on the ligand of Jagged1. Which ligands participate in this process needs to be further studied.

The potential role of NO in promoting renin secretion: NO is a short-lived, endogenously produced signaling molecule that plays multiple roles in mammalian physiology. NO is formed from its precursor l-arginine by a family of nitric oxide synthase (NOS), which has three identified isoforms: neuronal type NOS (nNOS), endothelial type NOS (eNOS), and inducible type NOS (iNOS). Different isoforms are expressed depending on the organs, tissues, and cells [75,76]. 

The role of NO in renin secretion under hypoxia is not clear, but a potential link between NO and renin expression under hypoxia is revealed by reviewing previous studies. In theory, NO can participate in both inhibitory and stimulatory pathways of renin secretion. NO can stimulate soluble guanylate cyclase (sGC) and increase cyclic guanosine monophosphate (cGMP). Then, cGMP-mediated protein kinase (PKG II) and phosphodiesterase (PDE) 2 are involved in the inhibition of renin secretion, whereas the cGMP-mediated inhibition of PDE3 prevents cyclic adenosine monophosphate (cAMP) degradation and stimulates renin secretion. Thus, NO can have dual effects on renin release. The controversial regulation ability of NO on renin secretion could be partly explained by different sources of NO. Increased NO from eNOS activation is always accompanied by elevated renal perfusion and shear stress, which is probably involved in the inhibition of renin release by the activation of PKG II. Another source of NO for JG cells is nNOS, which isinvolved in the activation of renin secretion by inhibiting PDE3 activity [77]. However, current research data have established that the overall effect of NO on renin secretion is stimulatory [14]. The increase in renin secretion observed in response to a low salt concentration was markedly attenuated in the presence of the nonspecific NOS inhibitor (NG-nitro-l-arginine). Similar results were obtained in vivo, where increased renin secretion in response to loop diuretics was attenuated by concomitant NOS inhibition. The availability of NO is also required for the recruitment of renin-expressing cells, in particular, for the recruitment of preglomerular VSMCs [78]. The promotion of NO on renin may be related to the source of NO under hypoxia. eNOS, nNOS, and eNOS all contribute to NO under hypoxia, but nNOS is the main source of NO under hypoxia [79]. NO derived from nNOS can stimulate cGMP, which further mediates the activation of renin secretion by inhibiting PDE3 activity [77]. 

Hydrogen sulfide (H2S) is an endogenously produced gas with known antioxidant and neuroprotective properties. H2S has a complex interaction with NO, which can upregulate eNOS expression, increase NO bioavailability by reducing oxidative stress, and enhance downstream NO signaling by inhibiting PDE5A activity [80]. Like NO, enhanced H2S production has been proposed as a universal response to hypoxic stress. The increase in H2S has an inhibitory role in the pathological signaling of RAS. H2S has been reported to downregulate cAMP by inhibiting adenylate cyclase activity, thereby regulating renin release [81,82,83]. 

Natriuretic peptides: Natriuretic peptides are hormones secreted from the heart to promote Na^+^ excretion in the kidneys. Currently, there are three known peptides in the natriuretic peptides family: atrial natriuretic peptide (ANP), B-type natriuretic peptide (BNP), and C-type natriuretic peptide (CNP) [84]. ANP is hypoxia-responsive and its circulating levels are increased by decreased clearance due to downregulation of the natriuretic peptide receptor-C in hypoxia [85]. The increased ANP has an inhibition role for renin secretion in JG cells. The inhibition role of ANP on renin may be related to an increase in cGMP [86]. Further, it was shown that the inhibitory effects of ANP on renin are mediated by the cGMP/PDE2 pathway, which promotes cAMP degradation [87].

## 6. Renin in Renal Local RASs under Hypoxia

In the classical definition, RAS is a peptidergic system with endocrine characteristics. Current results have changed our view of RASs and introduced the concept of “local” or “tissue” RASs. For inter-renal RASs, the observation of renin was reported in collecting ducts, proximal tubules and the bowman capsule. The inter-renal RAS also has a complex interaction with systemic RASs. Ang II produced in systemic RASs acts in a feed-forward manner to stimulate local renin synthesis. Furthermore, some components of local RASs, such as renin or angiotensinogen, may be taken up from the systemic RAS in circulation. Such local RASs and their interaction with the systemic RAS make the story of RAS complex [5].

Hypoxia occurs commonly in chronic kidney diseases because tubulointerstitial fibrosis impairs oxygen diffusion and supply [88]. The activation of the intrarenal RAS in the CKD model has been evaluated by immunoreactivities for Ang II in the tubulointerstitial area. The activation score of interstitial Ang II correlated with plasma creatinine concentration, glomerulosclerosis, fibrosis, and cell infiltration in interstitial inflammation [89]. UUO, characterized by tubulointerstitial fibrosis, showed increases in renal renin content, ACE activity, and Ang II concentration on the first day after surgery. Angiotensin II receptor antagonists ameliorate renal tubulointerstitial fibrosis caused by UUO, which suggests a pathogenic role of the intrarenal RAS in renal fibrosis [90,91]. NO also has a regulatory role in the local RAS, but the regulation is complicated. Curnow’s team found that different levels of NO bioavailability have different regulation roles in renin synthesis in the collecting duct: low NO bioavailability enhances the synthesis and secretion of renin in collecting duct; high level of NO promotes the accumulation of renin intracellularly, but does not increase renin secretion in collecting duct [92]. 

## 7. Future Prospects

In recent years, our understanding of the physiological and pathological activity of renin under hypoxia has gradually deepened. The results obtained from several related studies have shed light on questions regarding the change in renin under hypoxia. Both systemic and local renin show an increase in most hypoxia conditions. The increase in renin expression under hypoxia comes from the activation of JG cells and the recruitment of renin lineage cells. The possible regulation mechanisms of renin activity under hypoxia include HIF signaling, Notch signaling, NO, and natriuretic peptides. 

However, some questions remain unanswered. For example, renin expression, but not renin secretion, was reported in recruitment cells. A recent study also found that the re-expression of renin in recruitment cells cannot store renin. Thus, whether the re-expression of renin in recruitment cells is accompanied by renin secretion is still unknown. In addition, Notch signaling and NO-induced renin secretion under hypoxia are two potential mechanisms that may explain the increased activity of renin under hypoxia. We reviewed relevant articles and highlighted possible links, but no study directly confirms the role of Notch signaling and NO in renin secretion under hypoxia. Another problem is that evidence of decreased renin activity regulated by HIF signaling comes mainly from the HIF overexpression model under normoxia but not hypoxia. The question of whether HIF overexpression in normoxia can explain the change in renin activity in hypoxia remains to be further verified. Prorenin was previously considered to be the inactive precursor of renin, but recent findings show that prorenin has a more complex regulation on RAS via prorenin receptors. Related research about prorenin under hypoxia is still rare and studies in this field should be carried out in the future. This review focused on the change in kidney renin but did not cover the downstream of renin, like aldosterone or Ang-(1–7), because the downstream signaling of renin is complicated. For one thing, RAS is not limited to the classical axis. For another, the classical downstream aldosterone also has a renin-independent activation pathway. Current studies cannot tell us the full map of RAS under hypoxia. Thus, we expect future studies to provide us with the answers to these questions. 

## Data Availability

No new data were created or analyzed in this study. Data sharing is not applicable to this article.
